# A GMP-compliant manufacturing method for Wharton’s jelly-derived mesenchymal stromal cells

**DOI:** 10.1186/s13287-024-03725-0

**Published:** 2024-05-03

**Authors:** Wanglong Chu, Fen Zhang, Xiuping Zeng, Fangtao He, Guanyan Shang, Tao Guo, Qingfang Wang, Jianfu Wu, Tongjing Li, Zhen Zhong Zhong, Xiao Liang, Junyuan Hu, Muyun Liu

**Affiliations:** 1 Shenzhen Beike Biotechnology Co., Ltd, 518000 Shenzhen, Guangdong People’s Republic of China; 2 National Engineering Research Center of Foundational Technologies for CGT Industry, 518000 Shenzhen, Guangdong People’s Republic of China; 3 Shenzhen Kenuo Medical Laboratory, 518000 Shenzhen, Guangdong People’s Republic of China

**Keywords:** Enzymatic digestion, Good manufacturing practice, Mesenchymal stem cells, Scale-up, Stability, Wharton jelly

## Abstract

**Background:**

Wharton’s jelly-derived mesenchymal stem cells (WJ-MSCs) hold great therapeutic potential in regenerative medicine. Therefore, it is crucial to establish a Good Manufacturing Practice (GMP)-compliant methodology for the isolation and culture of WJ-MSCs. Through comprehensive research, encompassing laboratory-scale experiments to pilot-scale studies, we aimed to develop standardized protocols ensuring the high yield and quality of WJ-MSCs manufacturing.

**Methods:**

Firstly, optimization of parameters for the enzymatic digestion method used to isolate WJ-MSCs was conducted. These parameters included enzyme concentrations, digestion times, seeding densities, and culture media. Additionally, a comparative analysis between the explant method and the enzymatic digestion method was performed. Subsequently, the consecutive passaging of WJ-MSCs, specifically up to passage 9, was evaluated using the optimized method. Finally, manufacturing processes were developed and scaled up, starting from laboratory-scale flask-based production and progressing to pilot-scale cell factory-based production. Furthermore, a stability study was carried out to assess the storage and use of drug products (DPs).

**Results:**

The optimal parameters for the enzymatic digestion method were a concentration of 0.4 PZ U/mL Collagenase NB6 and a digestion time of 3 h, resulting in a higher yield of P0 WJ-MSCs. In addition, a positive correlation between the weight of umbilical cord tissue and the quantities of P0 WJ-MSCs has been observed. Evaluation of different concentrations of human platelet lysate revealed that 2% and 5% concentrations resulted in similar levels of cell expansion. Comparative analysis revealed that the enzymatic digestion method exhibited faster outgrowth of WJ-MSCs compared to the explant method during the initial passage. Passages 2 to 5 exhibited higher viability and proliferation ability throughout consecutive passaging. Moreover, scalable manufacturing processes from the laboratory scale to the pilot scale were successfully developed, ensuring the production of high-quality WJ-MSCs. Multiple freeze-thaw cycles of the DPs led to reduced cell viability and viable cell concentration. Subsequent thawing and dilution of the DPs resulted in a significant decrease in both metrics, especially when stored at 20–27 °C.

**Conclusion:**

This study offers valuable insights into optimizing the isolation and culture of WJ-MSCs. Our scalable manufacturing processes facilitate the large-scale production of high-quality WJ-MSCs. These findings contribute to the advancement of WJ-MSCs-based therapies in regenerative medicine.

**Supplementary Information:**

The online version contains supplementary material available at 10.1186/s13287-024-03725-0.

## Introduction

Mesenchymal stem/stromal cells (MSCs) were first discovered by Friedenstein in bone marrow in the 1970s [1] and named by Caplan in 1991 [2]. Pittenger demonstrated that MSCs could differentiate into adipocytic, chondrocytic, or osteocytic lineages [3]. The International Society of Cellular Therapy (ISCT) proposed the minimal criteria for defining MSCs in 2006, and in 2015, they suggested incorporating immune functional assays into MSC potency release criteria [4, 5].

Thus far, a large number of clinical trials involving MSC therapy have been conducted worldwide, the number of those registered on Clinicaltrial.gov has exceeded 10,000 cases (www.clinicaltrial.gov). Besides, approximately ten MSC-based cell therapy products have been approved for clinical use worldwide, such as Prochymal, Temcell, Alofisel, Cupistem, Stempeucel, and others [6]. Based on their immunomodulatory and tissue repair properties, MSCs have been used as a treatment for graft-versus-host disease (GvHD), systemic lupus erythematosus (SLE), inflammatory bowel disease (IBD), ischemic stroke (IS), Crohn’s disease (CD), knee osteoarthritis (KOA), spinal cord injury (SCI), and critical limb ischemia (CLI), among others [6–10].

To date, the predominant focus in clinical studies and product development has been on using MSCs derived from bone marrow (BM-MSCs) or adipose tissue (AT-MSCs). However, in recent years, significant attention has been directed toward WJ-MSCs as a valuable source of mesenchymal stem cells. The availability of WJ-MSCs is facilitated by their derivation from medical waste, typically discarded after birth, which ensures easy accessibility while minimizing pain and ethical concerns. Moreover, WJ-MSCs possess noteworthy characteristics, such as low immunogenicity and high proliferation capability, thus enabling large-scale expansion. Importantly, long-term in vitro culture of WJ-MSCs appears to have minimal impact on their phenotype and genetic stability. Clinical trials have not unveiled any long-term adverse effects or tumor formation associated with WJ-MSCs. This further bolsters their potential for a broad range of clinical applications [11–17].

Due to the significant potential of WJ-MSCs in regenerative medicine, extensive research has been conducted to optimize the manufacturing process [18, 19]. Efficient and reliable methods for WJ-MSC isolation from the umbilical cord are critical to harnessing their therapeutic potential. Currently, the main methods commonly used are the explant method and the enzymatic digestion method [20]. The explant method involves the cultivation of small tissue fragments from Wharton’s jelly to obtain MSCs, while the enzymatic digestion method utilizes specific enzymes to dissociate the matrix of umbilical cord tissues and release MSCs. Although the enzymatic digestion process carries a potential risk of damaging the cells if not performed under appropriate conditions, the explant method provides a simpler approach that minimizes external factors that could compromise cell viability and functionality, thus preserving the integrity of WJ-MSCs. However, it is important to note that standardizing the explant method can present challenges. Several studies have compared the two methods. While differences in primary cell culture time and yield can be observed, there are no significant disparities in terms of cell viability, morphology, proliferation, surface marker expression, and differentiation capacity after passaging. These findings suggest that both methods result in comparable outcomes for maintaining the desired characteristics of MSCs [21–26]. Apart from the WJ-MSCs isolation, several ongoing studies are focused on developing GMP-compliant production processes for clinical-grade WJ-MSCs, involving process design, manufacturing protocols, quality control, and characterization research [18, 27–31]. Nevertheless, despite reports on isolation methods and GMP-compliant production for WJ-MSCs have been available, there remains a dearth of in-depth research bridging the gap between laboratory-scale research, understanding of process parameters, and pilot-scale manufacturing.

The objective of this study was to develop a more precise and standardized GMP-compliant manufacturing method for isolating and culturing WJ-MSCs. This includes the selection of GMP-compliant reagents, investigation of various parameters involving the isolation and passaging process, and conducting extended passaging studies to determine the appropriate generations for clinical use. Moreover, translational studies were undertaken to transition from laboratory research to pilot-scale production. Additionally, several stability studies were conducted to assess the storage and utilization of the MSCs, aiming to determine the appropriate storage conditions and duration. Through comprehensive research, an optimized manufacturing process was established, ensuring that the isolated MSCs can be utilized as safe and effective therapeutic agents.

## Materials and methods

### UC tissue collection and preprocessing

Umbilical cord (UC) tissue was collected from Shenzhen Baoan District Maternity & Child Healthcare Hospital (Shenzhen, China). The collection process was approved by the Medical Ethics Committee of Shenzhen Baoan District Maternity & Child Healthcare Hospital. The screening, collection, transportation, and infectious disease testing of parturients were carried out according to our standard operating procedures (SOPs). In short, the mothers were between the ages of 20–35 and free from infectious diseases and family genetic diseases and provided written informed consent. After being collected following a cesarean section, the UC (> 20 cm length) was transported to our facility within 24 h at 2–10 °C. Both umbilical cord blood and maternal peripheral blood samples are subjected to pathogen testing, which includes HBV, HCV, HTLV, TP, HIV, EBV, and CMV.

Then, the UCs were weighed, rinsed, decontaminated, and divided into multiple segments. The cord length was estimated and the weight was measured. Subsequently, the cord was rinsed with DPBS (w/o Ca, Mg, Gibco™, USA) and decontaminated using a 0.5% povidone-iodine solution (ADF Hi-Tech Disinfectants, China) for 3 minutes. The cord was then rinsed with DPBS three times to ensure the removal of any remaining blood and disinfectant residue. Using a surgical scalpel, the UC was carefully cut into segments measuring 3–6 cm in length. These UC segments were opened to expose Wharton’s jelly and the underlying blood vessels. Next, two arteries and one vein were carefully removed, and the Wharton’s jelly was extracted. It was then rinsed again before being minced into 1–4 mm^3^ fragments. These fragments were weighed again and can be further utilized for the isolation of MSCs. The isolation can be performed using either the explant method, where small tissue fragments are directly placed onto the culture medium, or the enzymatic digestion method, which enzymatically breaks down the extracellular matrix to release desired cells for culture. The detailed process is described as follows.

### Enzymatic digestion optimization

To optimize the enzymatic digestion method for increased cell yield, various factors need to be taken into consideration, including the selection of enzymes, isolation parameters, and culture parameters. For application in clinical settings, the GMP grade enzyme Collagenase NB6 GMP (Nordmark Biochemicals, Germany) was recommended based on preliminary research conducted on various manufacturer brands. This optimized formulation includes collagenase class I and class II, as well as proteolytic activities such as neutral protease and clostripain. Isolating parameters primarily involve enzyme concentration, digestion time, temperature, and pH, while culture parameters focus on inoculation density after digestion and culture medium. It is worth noting that a temperature of 37 °C and a pH range of 7.0–7.4 have been identified as optimal for enzyme activity. Consequently, a comprehensive assay design was developed, encompassing varying enzyme concentrations (0.2, 0.4, 0.6 PZ U/mL) according to the manufacturer’s instructions, digestion times (2, 3, 4 h), seeding densities (0.5 g, 1 g, 2 g tissue per 75 cm^2^ flask), and culture mediums (MSC Serum- and Xeno-Free Medium (NutriStem®, Biological Industries, Israel) + 2% hPL (Stemulate®, Sexton Biotechnologies, USA), 5% hPL or 10% hPL). In the experiment, the seeding densities (g tissue per flask) represent the amount of tissue fragments used before digestion and then seeded into a specific size of the culture flask after digestion. We also measured the primary cell densities (cell number after digestion per flask) for analysis. Due to the complex composition of cell components immediately after digestion and significant variations among different samples, we selected post-cultured passage 0 (P0) generation cells as the evaluation indicator for the enzymatic digestion results.

In addition to these factors, the enzymatic digestion method generally follows the previously described basic procedure. Briefly, the tissue fragments were transferred to a bottle and twice more than the volume of collagenase media was added. The collagenase media was prepared by dissolving collagenase powder in DPBS to obtain the corresponding concentration (PZ U/mL). Then, the bottle was placed in a temperature-controlled shaking incubator for digestion, with digestion parameters set to 37 °C and 150 rpm. After digestion, the mixture was neutralized by adding three times the volume of DPBS. Filtration was then performed by passing the mixture through a 100-µm cell strainer (Falcon®, Corning, USA). Subsequently, centrifugation was carried out at 1000 g for 15 min to separate the suspended cells, the supernatant was removed, and the pellet was resuspended in DPBS. Another centrifugation step was performed at 400 g for 10 min to further remove collagenase residue. After discarding the supernatant, the pellet was resuspended in a culture medium. A sample was taken for cell counting (Vi-Cell Blu, Beckman Coulter Inc., USA), and the resulting cell suspension was transferred into a culture flask (Nunc™, ThermoFisher Scientific, USA) for incubation at 37 °C in a humidified atmosphere with 5% CO_2_. The first medium exchange was performed after 5 days, followed by subsequent medium exchanges every 3 days until days 10–15 or until the cell confluence reached 60-80%. These MSCs were harvested using recombinant trypsin (CTS™ TrypLE™ Select, Gibco™, USA) and designated P0. Cell morphology, quantity, viability, culture time, and immunophenotype were evaluated.

### Comparative analysis of the explant and enzymatic digestion methods

A head-to-head comparative study was conducted to analyze the differences between the explant method and the enzymatic digestion method. Briefly, 2 g UC tissue fragments were divided into two equal segments: one was directly added to the culture medium and transferred to 75 cm^2^ flasks, and the other underwent the collagenase digestion process as described above. WJ-MSCs were harvested at P0 and then seeded for continuous passage at a density of 4500–5500 cells per cm^2^. Cell morphology, cell count at P0, culture duration, immunophenotype, and population doubling time (PDT) of continuous passage were compared.

Additionally, the seeding density for continuous passage was optimized. Cells at P0 were subcultured at varying seeding densities of 1000 cells/cm^2^, 3000 cells/cm^2^, and 5000 cells/cm^2^ and cultured until P4. Culture time, PDT, and cumulative population doublings (CPDs) were analyzed. The concentrations of glucose and lactate in the culture supernatant were measured daily at P2 to compare metabolism at different seeding densities, using a glucose meter (Roche Diagnostics, Switzerland) and a lactate analyzer (EKA Diagnostics, Germany).

### Study of consecutive passaging WJ-MSCs

To assess the manufacturing process and determine the optimal passage number for clinical application, P0 cells obtained using the enzymatic digestion method were consecutively passaged up to P9 at a density of 4500–5500 cells per cm^2^. The evaluation included the change in cell morphology, PDT, cell viability, and cell diameter with different passages. CPDs were analyzed as well.

### Manufacture and scaling up from laboratory scale to pilot scale

All the above experiments were conducted on a laboratory scale based on culture flasks. To further evaluate whether the established method was suitable for GMP**-**compliant manufacturing, the production process was scaled up to a pilot scale based on a cell factory (Nunc™ EasyFill™ Cell Factory™, ThermoFisher Scientific, USA) and WJ-MSCs were passaged up to P5 (DP). A master cell bank (MCB) and working cell bank (WCB) were established at P1 and P3, respectively. The production and QC of three batches were carried out at the GMP-compliant manufacturing facility operated by Beike Biotechnology (Shenzhen, China).

In detail, first, According to the established isolation protocol, the cells were cultured in a 632 cm^2^ monolayer cell factory after enzymatic digestion of UC tissue fragments until the cell confluence reached 60-80%. Second, the P0 WJ-MSCs were harvested using recombinant trypsin, washed with resuspension media (DPBS and 5% human serum albumin (HSA) (HuaLan bio, China)) seeded into another monolayer cell factory at a density of 4500–5500 cells/cm^2^. After 3 days, when cell confluence reached 85 − 95%, the cells were harvested and cryopreserved to establish the master cell bank (P1). Third, the MCB cells were thawed and seeded into a monolayer cell factory at 4500–5500 cells/cm^2^, resulting in P2 cells. After a 3-day growth period, once cell confluence reached 85-95%, the P2 cells were subsequently harvested and seeded into a 4-layer cell factory to obtain P3 cells, which were cryopreserved to establish the working cell bank. Finally, the WCB cells were thawed and seeded into a monolayer cell factory, resulting in P4 cells at 4500–5500 cells/cm^2^. After 3 days, when cell confluence reached 85-95%, the P4 cells were then seeded into a 10-layer cell factory to obtain P5 cells, which were cryopreserved for further clinical use. The P1 cells were cryopreserved at a concentration of 5 × 10^6^ /mL, while the P3 and P5 cells were cryopreserved at a concentration of 1 × 10^7^ /mL. Cryopreservation was done using a solution consisting of 7.5% DMSO (Wak-Chemie Medical GmbH, Germany), 20% HSA, and 72.5% multiple electrolytes injection (KeLun Pharmaceutical, China). The cells were filled into AT-Closed Vial® (Aseptic Technologies, Belgium), and immediately frozen using a controlled-rate freezer with a freezing profile of − 1 °C/min. Subsequently, they were stored in the vapor phase of liquid nitrogen at temperatures below − 150℃.

### Stability of MCB, WCB, and DP

To validate the stability of pilot-scale production, an extensive series of studies were conducted. Firstly, the MCB, WCB, and DP were subjected to a long-term stability study, whereby they were stored at temperatures below − 150℃ for 0, 3, and 6 months. In addition, multiple freeze-thaw cycles of DP were performed, considering the potential clinical use scenarios. These cryopreserved cells were reanimated by immersing them in a 37 °C water bath. Subsequently, a portion of the cells underwent QC testing. The remaining cells were cryopreserved again as above. This freeze-thaw cycle was repeated three times, with QC testing carried out after each cycle. Furthermore, the in-use stability of DP was assessed. The procedure involved thawing the cells and diluting them with 50mL of 0.9% sodium chloride injection and 10% HSA. The DP was then stored in a drug stability testing chamber with temperatures at 2–8℃ in the dark, or at 20–27℃ with an illuminance of 4500 ± 500 lx. This evaluation was conducted over 0, 2, 4, and 8 h. The QC evaluation encompassed assessing changes in cell viability, cell quantity, expression of cell surface markers, mixed lymphocyte reaction (MLR), and microbial safety testing.

### Quality control assays

#### Cell morphology

During the initial day of separating UC-derived MSCs, each culture medium exchange and cell harvest, cell observations were performed using an inverted microscope (IX73, Olympus, Japan). These observations encompassed cell morphology and cell confluence analysis.

#### Cell counting and viability

The cell numbers and viability were determined by an automatic cell counter (Vi-Cell Blu, Beckman Coulter, Inc., USA) with the trypan blue exclusion method. After mixing Trypan Blue dye with the cell suspension and placing it into the cell counter, the counter captured 100 images for the analysis of cell count, viability, and cell diameter (µm).

#### Cell proliferation analysis

Cell proliferation analysis included calculating the population doubling time (PDT) and population doubling level (PDL). PDT was calculated by the formula X = T × log2 / (logN - logX_0_), where T is the time between initial plating and harvest for the respective passage, N is the total number of harvested cells, and X_0_ is the total number of initial plating [32, 33]. PDL was calculated by the formula X = (logN - logX_0_) / log2 [34].

#### Immunophenotype

Surface antigen phenotyping of WJ-MSCs was assessed using flow cytometry (FACSCalibur™, BD Biosciences, USA) and analyzed with CellQuest Pro software. The cells were stained with anti-human antibodies labeled with phycoerythrin (PE), allophycocyanin (APC), fluorescein isothiocyanate (FITC), or PerCP. The specific antibodies used for staining included CD73-PE, CD44-FITC, CD29-PE, CD166-PE, CD45-FITC, CD34-PE, CD14-FITC, CD79a-APC, HLA-DR-PerCP (BD Pharmingen™, BD Biosciences, USA), CD105-APC (eBioscience™, ThermoFisher Scientific, USA), CD90-FITC, CD31-PE, HLA-ABC-APC, CD80-FITC, CD40-PE, and CD86-APC (Biolegend®, BioLegend Inc., USA). Before and following staining, samples were washed with 1× PBS. Isotype antibodies from the same manufacturers for mice or rats were used as controls.

#### Growth curve and cell cycle assay

WJ-MSCs were initially seeded at a density of 10,000 cells per 12-well plate (Falcon®, Corning, USA). Starting from Day 1 and continuing until Day 7, cells were harvested from three wells every day and counted using an automatic cell counter (Vi-Cell Blu, Beckman Coulter, Inc., USA). The cell counts obtained during the logarithmic growth phase were utilized to calculate the PDT using the listed formula. Further, on Day 3, WJ-MSCs were harvested specifically for the cell cycle assay. The Cycletest™ Plus DNA Kit (BD Biosciences, USA) was employed to determine the cell cycle, and the assay was conducted according to the manufacturer’s instructions. The proliferative index (PI) was calculated by the formula PI= (S + G2/M)/(G0/G1 + S + G2/M) [35].

#### CFU-F assay

WJ-MSCs were initially seeded at a density of 100 cells per 6-well plate (Falcon®, Corning, USA) in triplicate. After culturing for 10–14 days, the cells were washed with 1× PBS, fixed in 100% methanol for 30 min, stained with 0.1% crystal violet for 45 min, and then rinsed in tap water 2–3 times. Aggregates consisting of 50 cells or more were defined as CFU-Fs (colony-forming units-fibroblasts). The data are reported as the total number of colonies per 100 cells [36–37].

#### Cellular senescence assay

The cellular senescence assay was performed using a senescence β-galactosidase staining kit (Beyotime Biotechnology, China) following the manufacturer’s instructions. Briefly, WJ-MSCs were seeded in a 6-well plate at a density of 2000–3000 cells and cultured for 72 h. Subsequently, the cells were washed with 1× PBS, fixed at room temperature for 15 min, and then washed thrice with 1× PBS. Following this, the cells were incubated overnight at 37 °C in a dry incubator with the X-gal staining mixture. After washing away the staining solution, the cells were visualized and captured using an inverted microscope (CKX53, Olympus, Japan). Cells displaying a distinct blue staining were recognized as senescent cells. Five images were captured at random fields within each well, with a total of four wells per sample. Representative fields were visualized using a 100× magnification. The number of senescent cells and the total cell count were quantified from the images. The ratio of senescent cells to the total cell count represents the percentage of cellular senescence.

#### Multilineage differentiation

The differentiation potential of WJ-MSCs was assessed using the OriCell® osteogenic, adipogenic, and chondrogenic differentiation Kit (Cyagen Biosciences Inc., China) according to the manufacturer’s instructions. Briefly, for osteogenic and adipogenic differentiation, the cells were cultured in a 6-well plate at 37 °C with 5% CO_2_ until reaching approximately 70-100% confluence. Subsequently, the culture medium was replaced with osteogenic or adipogenic differentiation medium and exchanged every 3 days. After 2 to 4 weeks of incubation, the cells were stained with Alizarin Red S solution or Oil Red O solution to evaluate osteogenic or adipogenic differentiation, respectively. For chondrogenic differentiation, (3–4) × 10^5^ cells were used to differentiate into chondrogenic cells for a period of 3 to 4 weeks, which were then stained with Alcian Blue solution.

The stained images were analyzed under an inverted microscope (100 × magnification; CKX53, Olympus, Japan). In each differentiation assay, cells grown in the regular medium were used as the negative control.

#### Karyotype analysis

Karyotyping was conducted using the Giemsa stain technique. First, cell division was halted in metaphase with 0.3 µg/mL colchicine (Solarbio, China) at 37 °C for 2–3 h. Afterward, the cells were washed and trypsinized, suspended in a warmed hypotonic solution (0.075 M KCl), and incubated for 30–40 min at 37 °C. Following incubation, the cells were washed with a fixative solution consisting of a mixture of methanol and glacial acetic acid at a 3:1 ratio 3 times. Subsequently, the cells were resuspended in a fresh fixative solution and dropped onto clean slides, which were placed in ice water. To obtain G-bands, the slides were dried at a temperature of 70 °C for a minimum of 3 h. Next, the slides were immersed in a 0.01% trypsin solution for 3 min. They were then rinsed twice with saline solution before being stained using a 1:20 dilution of Giemsa solution (Bio Basic Inc., Canada). Evaluation of the band quality was performed under a microscope with a magnification set at 100×. Mitoses were captured using specialized software, and a minimum of 20 metaphases were analyzed in each sample.

#### Mixed lymphocyte reaction

The mixed lymphocyte reaction (MLR) assay was performed following the protocol described by K. Zhang et al. [38]. In brief, peripheral blood mononuclear cells (PBMCs) were thawed and suspended in PBS and labeled with carboxyfluorescein diacetate succinimidyl ester (CFSE) (Solarbio, China). The CFSE-labeled PBMCs were stimulated with 10 ng/ml PHA (Beyotime Biotechnology, China) and cocultured with hWJ-MSCs (5:1) at 37 °C and 5% CO_2_ in a 12-well plate for 4–5 days. Following the incubation period, all lymphocytes were collected, and cell proliferation was determined by monitoring the gradual reduction in CFSE fluorescence using a BD FACS Calibur flow cytometer. PBMCs without labeling, PHA stimulation, or coculturing were used as negative controls.

#### Microbial safety test

Microbial safety testing was conducted following the guidelines set third in the Pharmacopoeia of the People’s Republic of China (ChP). The bacterial and fungal tests employed a culture method, while the mycoplasma test utilized both the culture method and the indicated cell culture method. The endotoxin test was performed using the Limulus amebocyte lysate method.

### Statistical analysis

Statistical analyses and graphs were performed using GraphPad Prism 8.0. Data are expressed as the mean ± standard deviation (SD). Significant differences between groups were assessed by paired t-tests. A *p*-value less than 0.05 was deemed to have statistical significance. The correlation between the grams of Wharton’s jelly, the quantities of primary cells after digestion, and the quantities of P0 WJ-MSCs were assessed using linear regression analyses. The coefficient of determination (R^2^) and p-value were used to evaluate the goodness of fit. A *p*-value less than 0.05 is considered statistically significant, indicating a strong association between the variables.

## Results

### Enzymatic digestion optimization

The parameters studied in this research included enzyme concentrations (0.2, 0.4, 0.6 PZ U/mL), digestion times (2, 3, 4 h), seeding densities (0.5 g, 1 g, 2 g tissue per 75cm^2^ flask), and culture mediums (2%, 5%, 10% hPL). UCs were collected from a total of 9 donors; 3 donors’ UCs were utilized for studying enzyme concentrations and digestion times, another 3 were used for investigating seeding densities, and the remaining 3 were used for culture medium studies.

**Fig. 1:**
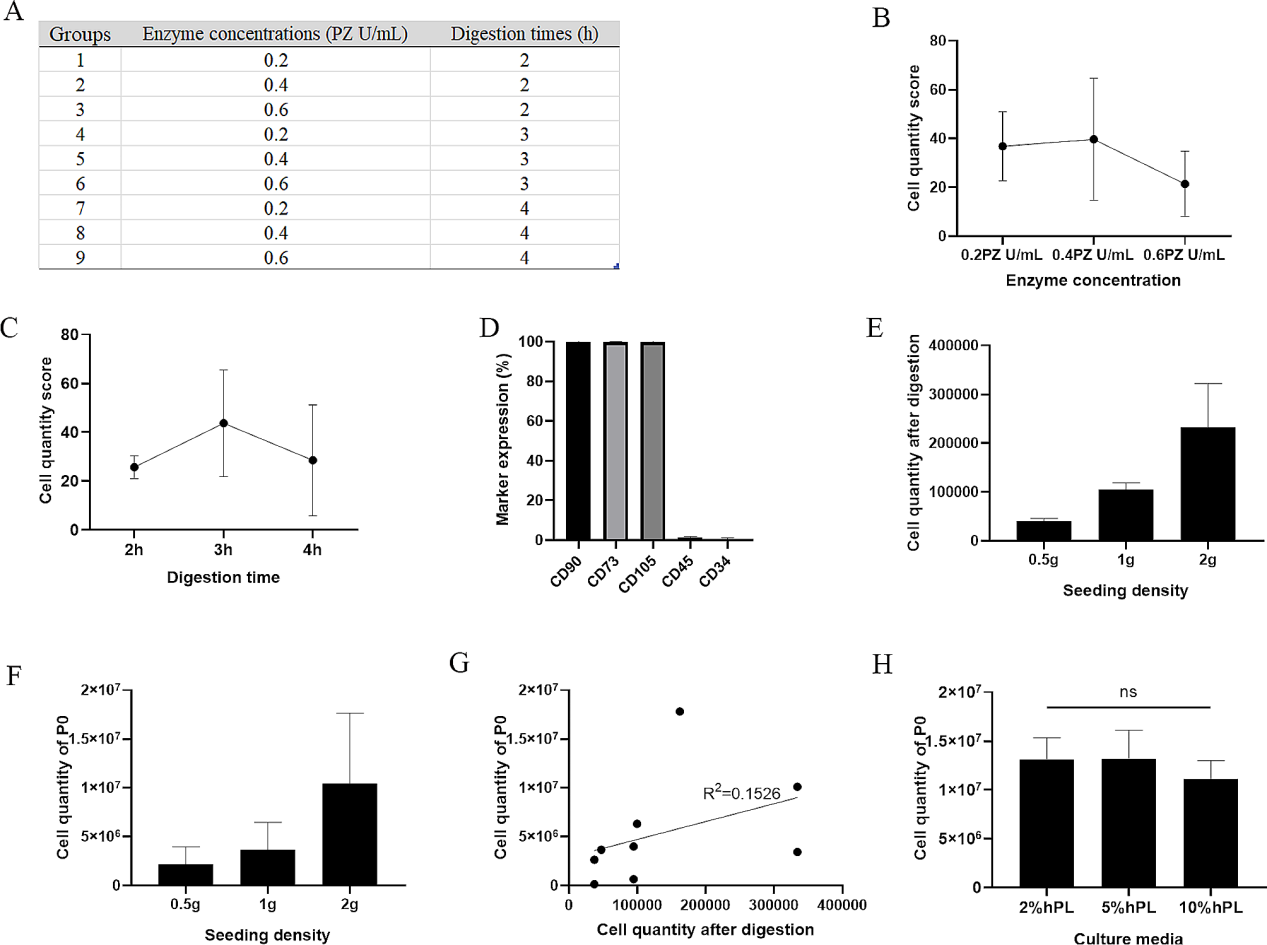
Enzymatic digestion optimization. (**A**) Experimental design of enzyme concentrations and digestion times. (**B-C**) Average cell quantity scores for 3 different enzyme concentrations and 3 digestion times. (**D**) Expression of surface markers on P0 WJ-MSCs obtained through the enzyme digestion method. (**E-F**) Correlations between the weight of Wharton’s jelly and the quantities of primary cells after digestion, as well as the quantities of P0 WJ-MSCs. (**G**) Correlations between the primary cell quantities and P0 WJ-MSCs quantities in different samples. (**H**) Effects of hPL concentrations on P0 WJ-MSCs quantities. P0 represents passage 0. P0 WJ-MSCs refer to the initial passage of umbilical cord-derived mesenchymal stem cells obtained through the described experimental conditions. ns = not significant

An orthogonal experimental design was employed to investigate the effects of enzyme concentrations and digestion times, resulting in nine groups of data in each donor’s UC (Fig. 1A). After digestion with collagenase and culture for 10–15 days, P0 WJ-MSCs were harvested. The mean quantities of P0 WJ-MSCs in three donor’s UC were found to be 1.83 × 10^6^ ± 1.30 × 10^6^, 5.86 × 10^5^ ± 5.39 × 10^5^, and 6.66 × 10^5^ ± 8.78 × 10^5^, respectively. Notably, the standard deviations indicate significant differences among the nine groups. To facilitate better comparison, we transformed the cell quantities into scores. A group of each sample was assigned a maximum score of 100 based on the highest cell quantity observed. The remaining groups were then proportionately adjusted to derive their respective scores in each group. The average cell score was calculated for each parameter, as illustrated in Fig. 1B-C. Concentrations at 0.4 PZ U/mL led to an observed increase in cell quantity, while concentrations at 0.6 PZ U/mL showed a decreasing trend. Similarly, digestion times of 3 h were associated with an increase in cell quantity, whereas digestion times of 4 h exhibited a declining trend. The expression of surface markers on the P0 WJ-MSCs was consistently stable across different samples and groups (Fig. 1D).

In addition to the digestion parameters, the culture parameters also influence the quantities of P0 WJ-MSCs. After digesting 0.5, 1, and 2 g of Wharton’s jelly, primary cells were obtained in amounts of 4.05 × 10^4^ ± 5.89 × 10^3^, 1.05 × 10^5^ ± 1.40 × 10^4^, and 2.32 × 10^5^ ± 9.03 × 10^4^, respectively. Subsequently, the quantities of P0 cells generated were 2.14 × 10^6^ ± 1.82 × 10^6^, 3.64 × 10^6^ ± 2.84 × 10^6^, and 1.15 × 10^7^ ± 7.22 × 10^6^, respectively. A positive significant linear correlation was observed between the grams of Wharton’s jelly used and the primary cells, as indicated by the high coefficient of determination (R^2^ = 0.7723, *p* < 0.005). Correspondingly, the quantities of P0 WJ-MSCs generated also exhibited a linear relationship with the grams of the initial Wharton’s jelly used (R^2^ = 0.4690, *p* < 0.05) (Fig. 1E-F). Nevertheless, no correlation was found between the quantities of primary cells and P0 WJ-MSCs among the different samples (R^2^ = 0.1526, *p* > 0.05), as shown in Fig. 1G.

Interestingly, it was observed that the addition of 2% hPL had an equivalent effect to that of 5% hPL on the quantities of P0 WJ-MSCs obtained. In contrast, increasing the hPL concentration further to 10% resulted in a decrease in the quantity (Fig. 1H). Nevertheless, no significant differences were observed (*p* > 0.05).

### Comparative analysis of the explant and enzymatic digestion methods

**Fig. 2:**
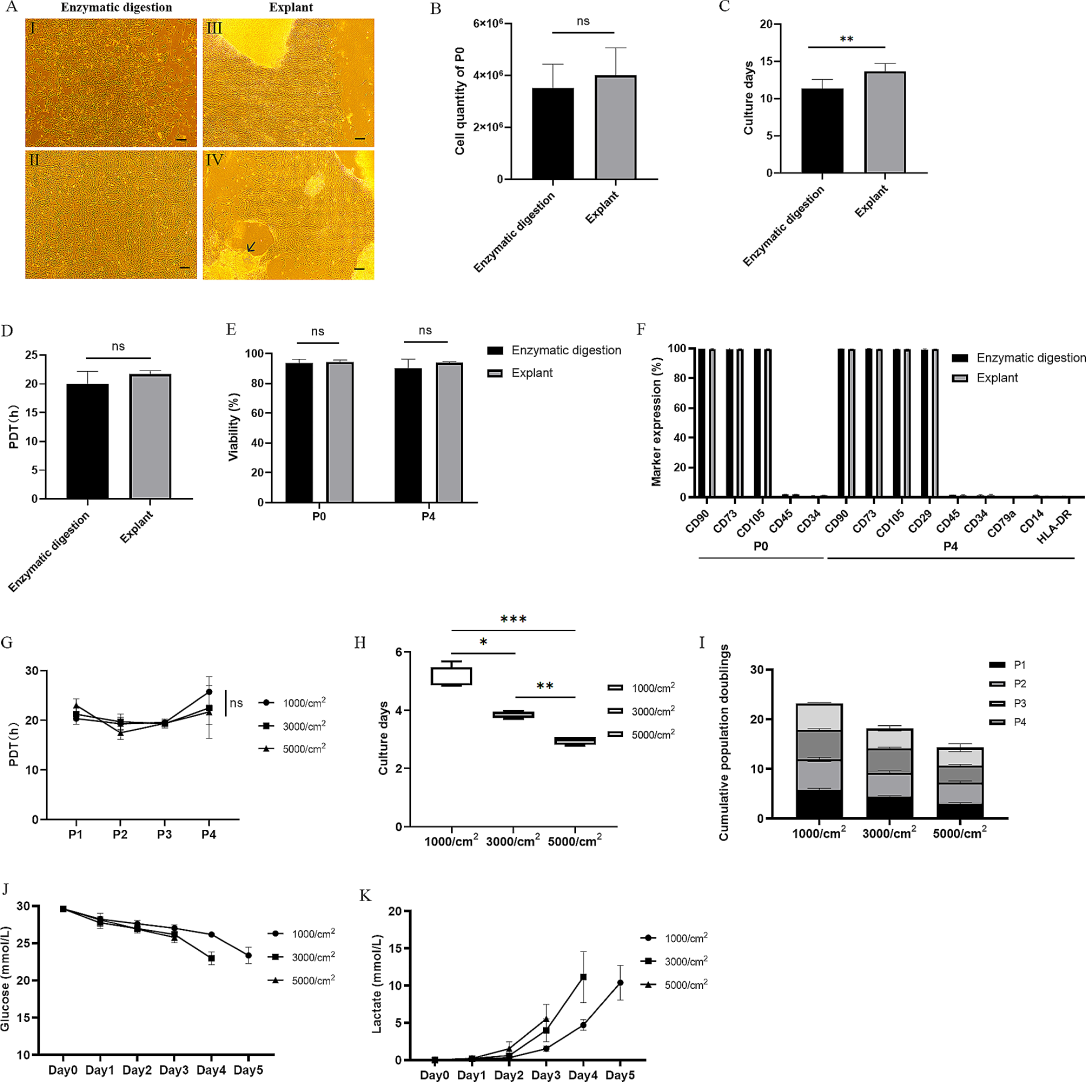
Comparison of enzymatic digestion and explant methods and optimization of the passaging density. (**A**) Representative images of P0 WJ-MSCs obtained through both enzymatic digestion (I-II) (left) and explant methods (III-IV) (right). The arrow indicated exfoliated cells. Bar = 200 μm. (**B-C**) Comparison of the quantity and culture duration of P0 WJ-MSCs harvested per gram of Wharton’s jelly between enzymatic digestion and explant methods. (**D**) Comparison of the average population doubling time (PDT) during passaging from P1 to P4 WJ-MSCs. (**E-F**) Comparison of cell viability and immunophenotypes of P0 and P4 WJ-MSCs. (**G**) Population doubling time (PDT) (**H**) and average culture duration (**I**) and cumulative population doublings (CPD) of cells at different seeding densities of 1000 cells/cm^2^, 3000 cells/cm^2^, and 5000 cells/cm^2^ from P1 to P4. (**J-K**) Daily changes in glucose and lactate concentration at P2 across different seeding densities. ^*^p < 0.05,^**^p < 0.01, ^***^p < 0.001, ns = not significant

Three UCs were collected, and the Wharton’s jelly obtained from each sample was separated into two groups. One group was used for collagenase digestion, while the other group was used for explant culture. The seeding density for both methods was 1 gram per 75cm^2^ flask. The enzymatic digestion method initiated cell proliferation between days 6–8 and exhibited uniform growth across the entire culture surface, while the explant method initiated cell proliferation between days 8–10 and demonstrated high-density growth in the center of the tissue, extending outward. It is noteworthy that exfoliated cells were observed before cell collection in the explant method culture. Besides, both types of P0 WJ-MSCs displayed a spindle-shaped morphology (Fig. 2A).

The average culture duration for P0 cells obtained through the enzymatic digestion method was 11.37 ± 1.21 days, significantly shorter than the 13.66 ± 1.06 days for the explant method (*p* < 0.01). The P0 WJ-MSCs harvested through the enzymatic digestion method yielded 3.53 × 10^6^ ± 9.02 × 10^5^ cells per gram of Wharton’s jelly, slightly lower but not significantly different from the explant method’s yield of 4.01 × 10^6^ ± 1.06 × 10^5^ cells (*p* > 0.05). Therefore, the enzymatic digestion method demonstrated faster cell proliferation due to a shorter culture duration (Fig. 2B-C).

No significant differences were observed in the average PDT during passaging from P1 to P4 or in the viability and immunophenotype of P0 and P4 WJ-MSCs between the two different methods (*p* > 0.05) (Fig. 2D, E, F). The PDT values ranged from 17.80 to 22.31 h among different samples, and the viability of P0 and P4 WJ-MSCs was above 85%. The expression levels of positive markers were consistently above 95%, while the negative markers were consistently below 2% for both.

When employing varying seeding densities for cell passages from P1 to P4 obtained from the enzymatic digestion method, no significant difference in the average PDT was observed (*p* > 0.05) (Fig. 2G). On the other hand, the culture duration at a density of 5000 cells/cm^2^ (2.96 ± 0.13 days) was significantly shorter than that at 3000 cells/cm^2^ (3.87 ± 0.11 days) (*p* < 0.01), and at 3000 cells/cm^2^ compared to 1000 cells/cm^2^ (5.08 ± 0.46 days) (*p* < 0.05) (Fig. 2H). Also, a significant difference was observed in CPDs passed to P4 at various densities (1000 cells/cm^2^ vs. 3000 cells/cm^2^ and 3000 cells/cm^2^ vs. 5000 cells/cm^2^, *p* < 0.01; 1000 cells/cm^2^ vs. 5000 cells/cm^2^, *p* < 0.001). Interestingly, CPDs of cells passed to P4 (14.28 ± 1.10) at 5000 cells/cm^2^ were similar to those passed to P3 (14.16 ± 0.81) at 3000 cells/cm^2^. Likewise, the cumulative PDT of cells passed to P4 (18.17 ± 1.34) at 3000 cells/cm^2^ approximated those of cells passed to P3 (17.91 ± 0.75) at 1000 cells/cm^2^, suggesting comparable cumulative total cell yields (Fig. 2I).

The results showed that when cells were cultured at 5000/cm^2^, cells consumed less glucose and accumulated less lactate compared to densities of 1000 cells/cm² and 3000 cells/cm², a difference that was statistically significant (*p* < 0.05) (Fig. 2J-K). On the 4th day at 3000/cm^2^, notably higher rates of glucose consumption (3.20 ± 0.10 mmol/L/day) and lactate accumulation (7.17 ± 2.26 mmol/L/day) were observed. In summary, a seeding density of 5000/cm^2^ resulted in a shorter culture duration and lower levels of glucose consumption and lactate accumulation.

Based on the findings, the enzymatic digestion method and a seeding density of 5000 cells/cm^2^ for continuous passage were chosen for the subsequent experiments.

### Study of consecutive passaging WJ-MSCs

**Fig. 3:**
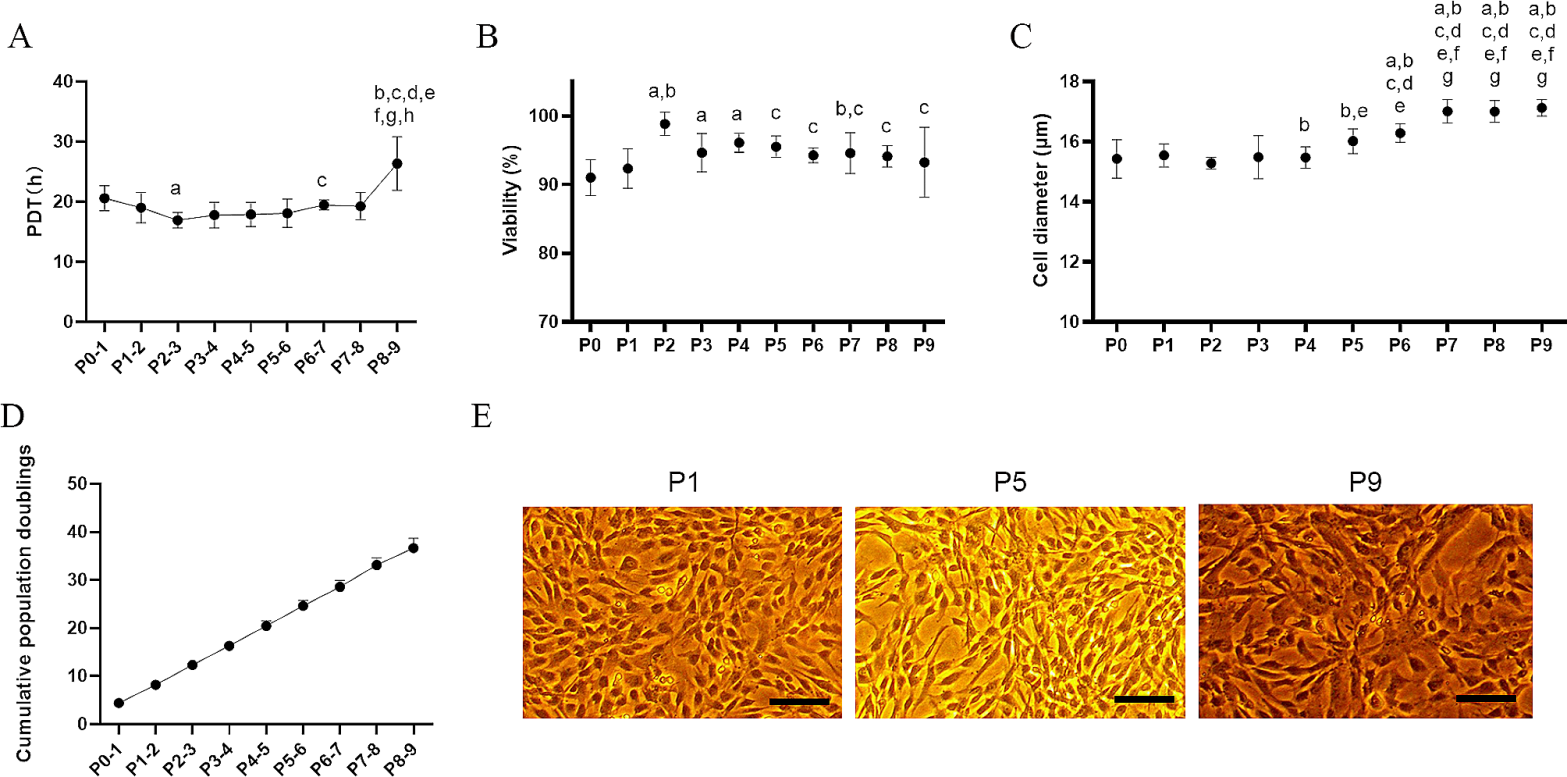
Characteristics of consecutive passaging WJ-MSCs (from P0 to P9) obtained using the enzymatic digestion method. (**A**) Comparison of population doubling time (PDT) (**B**) cell viability (**C**) and cell diameter among consecutive passages of WJ-MSCs. (**D**) Incremental increase in cumulative population doublings (CPDs) with each passage of WJ-MSCs. (**E**) Representative images of WJ-MSCs at different passages (P1, P5, and P9). In Figure A, significant differences between specific groups are indicated as a, b, c, d, e, f, g, h, corresponding to the comparisons P0-1, P1-2, P2-3, P3-4, P4-5, P5-6, P6-7, and P7-8, respectively. In Figures B and C, significant differences between specific groups and P0, P1, P2, P3, P4, P5, and P6 are denoted as a, b, c, d, e, f, and g, respectively. The notation a, b, c, d, e, f, g, h indicates significance at *p* < 0.05

To evaluate the activity of WJ-MSCs, an additional total of five UCs were collected, and the cells were continuously passaged up to P9. The PDT of the cells remained within the range of 15–30 h, gradually decreasing from P0 to P3, with P3 showing the shortest doubling time (16.92 ± 1.26 h) and the fastest cell proliferation. Significant differences were found when comparing P3 with P1, P7, and P9 (*p* < 0.05). However, starting from P6, the doubling time gradually increased, indicating a slowdown in cell expansion beyond the sixth passage (Fig. 3A).

The cell viability among different passages remained relatively consistent with minor variations, consistently exceeding 85%. Notably, the cell viability was higher in P2 (98.82% ± 1.70%) than in the other groups. Statistical significance (*p* < 0.05) was observed when comparing P2 with other groups, except for P3 and P4, showing the best cell activity during P2 (Fig. 3B).

The cell diameter ranged from 14.58 to 17.60 μm among different passages. From P5, the cell diameter gradually increased. Each passage from P0 to P4 cells showed significant differences compared to P6 (*p* < 0.05), as did each passage from P0 to P6 when compared to each passage from P7 to P9 (*p* < 0.05) (Fig. 3C).

With each passage, there was a consistent and incremental increase in CPDs, demonstrating continuous and reliable cell proliferation. The CPDs were recorded as 20.42 ± 1.05 at P5 and further increased to 36.66 ± 1.99 upon reaching P9 (Fig. 3D). The average population doublings (PDs) across all passages were 4.07 ± 0.54.

Although all cell passages exhibited a spindle-shaped morphology, notable differences were observed. P1 displayed a similar morphology to P5, with cells appearing small, short, and thin. In contrast, P9 cells appeared larger, longer, and more voluminous (Fig. 3E).

Therefore, P2 to P5 demonstrated superior cell activity, highlighting their potential clinical utility.

### Manufacture and scaling up from laboratory scale to pilot scale

**Fig. 4:**
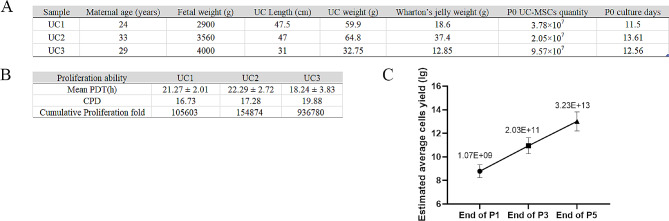
Proliferation analysis of WJ-MSCs in the scale-up study. (**A**) Information on umbilical cord (UC) samples. (**B**) Mean population doubling time (PDT), cumulative population doubling (CPD), and proliferation fold of WJ-MSCs cultured using the cell factories across all passages. (**C**) The estimated average quantities of WJ-MSCs at different passages (P1, P3, and P5) using cell factories

The scale-up study from culture flasks to cell factories was conducted on three UC samples. The UC sample information and Wharton’s jelly weight used in this study are shown in Fig. 4A. WJ-MSCs were isolated using the enzymatic digestion method. After being cultured for 10–15 days, the quantities of P0 WJ-MSCs obtained from each sample using the monolayer cell factory were determined to be 3.78 × 10^7^, 2.05 × 10^7^, and 9.57 × 10^7^ cells, respectively.

In Fig. 4B, the mean PDT, CPD, and proliferation fold of all passages cultured using the cell factory are presented. Notably, UC3 showed remarkable proliferation ability, with an average PDT of 18.24 ± 3.83 h across all passages and a proliferation fold reaching 936,780 from P0 to P5. As shown in Fig. 4C, the estimated average quantities of WJ-MSCs were projected to be 1.07 × 10^9^ at P1, 2.03 × 10^11^ at P3, and 3.23 × 10^13^ at P5, calculated by multiplying the P0 cells by the corresponding cumulative proliferation fold in each UC and then taking the average. Also, when divided by the weight of each UC, the estimated average quantities of WJ-MSCs per gram at P5 was 9.51 × 10^11^.

**Fig. 5:**
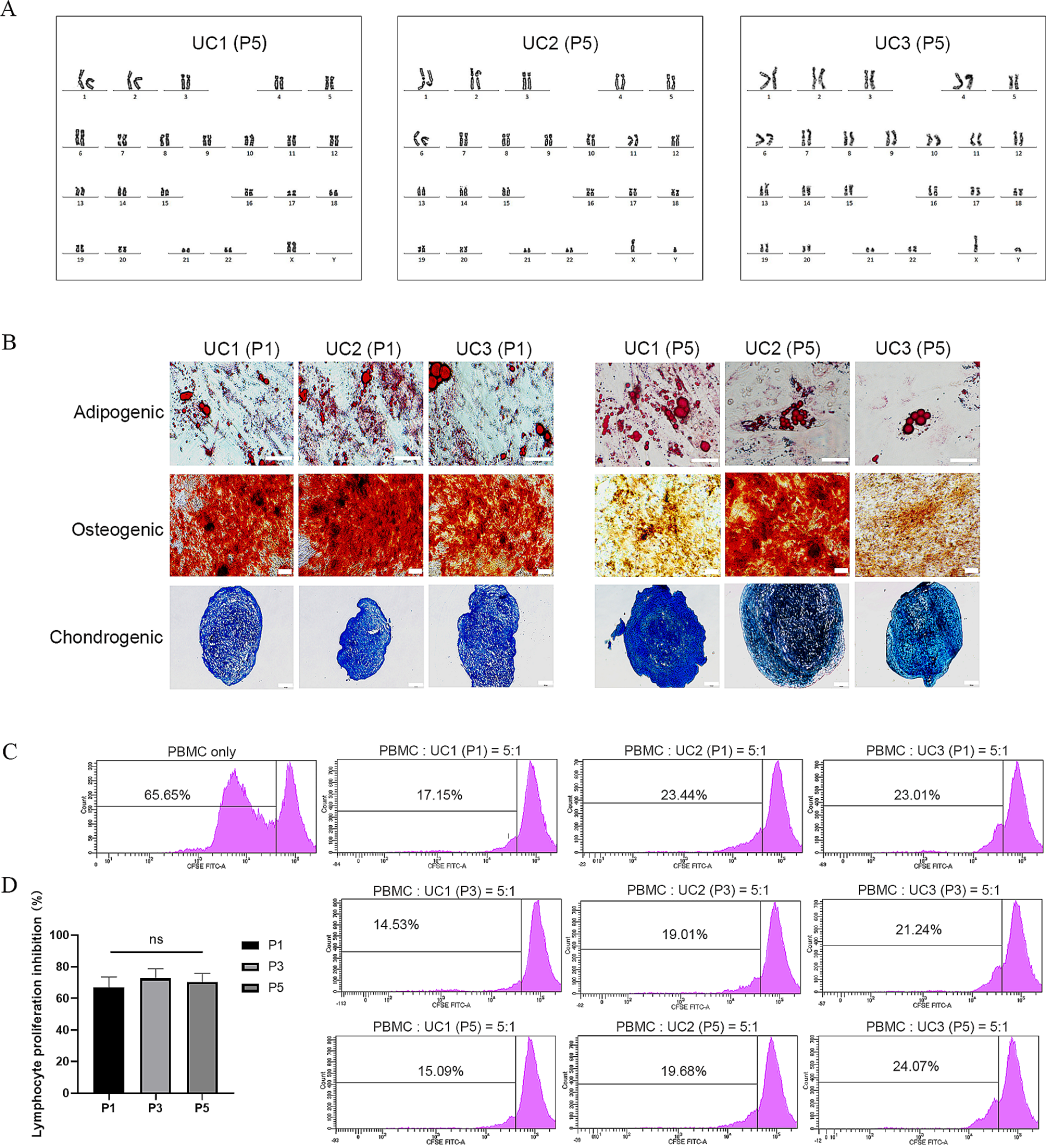
Quality control assessments in the scale-up study. (**A**) Cell viability percentages of WJ-MSCs from P0 to P5. (**B-C**) Expression levels of surface markers (CD73, CD90, CD105, CD44, CD29, CD166, CD45, CD34, CD79a, CD14, CD31, HLA-DR, HLA-ABC, CD80, CD40, CD86, and CD146) on WJ-MSCs at P1, P3, and P5. (**D-E**) Growth curves of WJ-MSCs at P1, P3, and P5 over 7 days. The mean population doubling time (PDT) was calculated from the growth curves. (**F-G**) Cell cycle percentage of G0/G1, S, and G2/M cells at P1, P3, and P5 after culture for 3 days. The proliferative index (PI) of WJ-MSCs at P1, P3, and P5 was obtained from cell cycle assays. (**H**) Number of colony-forming units formed by WJ-MSCs at P1, P3, and P5. (**I**) Cellular Senescence percentage of WJ-MSCs at P1, P3, and P5. ^*^p < 0.05

Quality control (QC) assessments were conducted on P1 (MCB), P3 (WCB), and P5 (DP), encompassing a range of evaluations, such as cell viability, immunophenotype, growth curve, cell cycle assay, CFU-F assay, cellular senescence assay, karyotype analysis, multilineage differentiation potential, MLR, and microbial safety testing. The viability was 94.60 ± 3.23% for P1, 89.70 ± 2.95% for P3 and 87.32 ± 0.81% for P5, with all values above 85% (Fig. 5A). The expression levels of CD73, CD90, CD105, CD44, CD29, and CD166 markers were uniformly greater than 95%, indicating positive immunophenotype characteristics. Conversely, the expression of CD45, CD34, CD79a, CD14, CD31, and HLA-DR markers was less than 2%, demonstrating the minimal presence of non-MSC contaminants. HLA-ABC exhibited positive expression, with P5 samples showing expression levels exceeding 95%, while P1 and P3 displayed expression levels above 60%. The costimulatory molecules CD80, CD40, and CD86 exhibited low expression levels below 5%, with CD80 and CD40 showing minimal expression and CD86 being relatively higher than CD80 and CD40 (*p* < 0.05 at P5). The expression of the therapeutic function-related marker CD146 was moderate and varied among the three samples, showing a decrease with an increase in passages (Fig. 5B-C).

To further evaluate the activity and growth potential of WJ-MSCs, several assays were conducted, including growth curve, cell cycle, CFU-F, and cellular senescence assays. The mean PDT calculated from the growth curve was 28.42 ± 1.46 h for P1, 26.70 ± 1.87 h for P3, and 28.98 ± 3.45 h for P5 (Fig. 5D-E). The PI calculated from the cell cycle assay indicated percentages of 48.51 ± 4.62% for P1, 48.34 ± 0.34% for P3, and 41.66 ± 5.16% for P5 (Fig. 5F-G). Additionally, the CFU-F assay revealed the formation of 21.78 ± 4.84 colonies for P1, 16.67 ± 7.51 colonies for P3, and 17.67 ± 7.57 colonies for P5 (Fig. 5H). Furthermore, the percentage of cells showing β-galactosidase activity, an indicator of cell senescence, was found to be 0.23 ± 0.19% for P1, 0.14 ± 0.11% for P3, and 0.56 ± 0.26% for P5. In comparison to P1 and P3, a significant decline in the percentage of cell senescence was observed in P5 (*p* < 0.05) (Fig. 5I). These results indicated overall favorable cell activity and growth potential in our study, albeit with a gradual decline observed as passages increased.

**Fig. 6:**
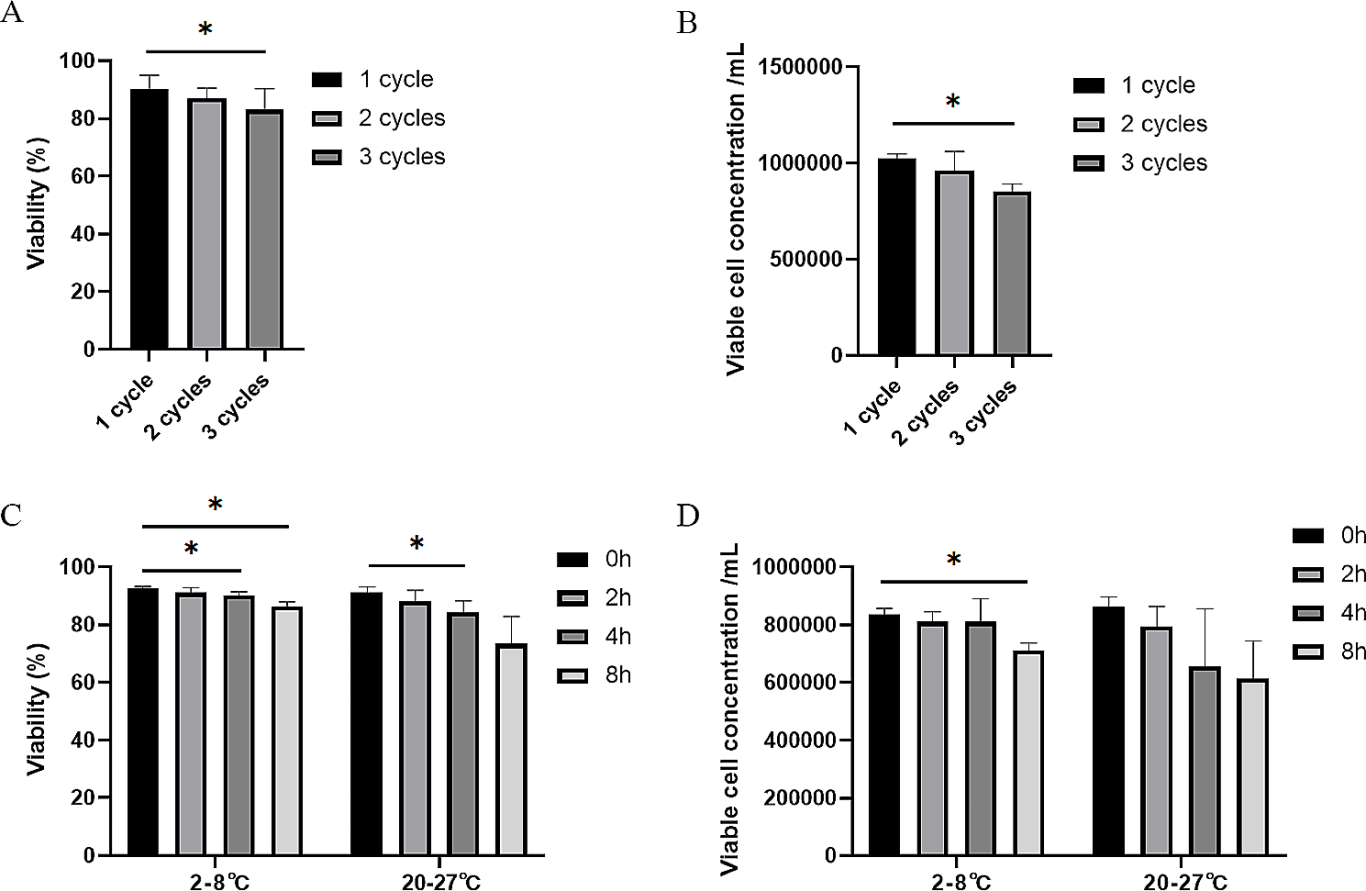
Safety analysis and biologic activity assay in the scale-up study. (**A**) Karyotype analysis of WJ-MSCs at P5. (**B**) Multilineage differentiation potential of WJ-MSCs at P1 and P5. (**C-D**) Mixed lymphocyte reaction assay of WJ-MSCs at P1, P3, and P5 to inhibit lymphocyte proliferation. ns = not significant

Karyotype analysis was performed at P5 to assess the genetic stability of WJ-MSCs. The results showed no numerical or structural chromosome abnormalities (Fig. 6A). In addition, microbial safety tests conducted on P1, P3, and P5 samples, including evaluations for bacteria, fungi, mycoplasma, and endotoxin, all yielded negative results. These findings are of significant importance in the context of GMP manufacturing.

In addition to the aforementioned analyses, a biological activity assay was conducted in this study. The P1 and P5 WJ-MSCs demonstrated multilineage differentiation potential, successfully differentiating into osteogenic, adipogenic, and chondrogenic lineages. Nevertheless, the findings indicate a potential decline in osteogenic differentiation capacity in UC1 and UC2 at P5 compared to P1, as illustrated in Fig. 6B. In addition, MLR was performed to evaluate the inhibitory effect of WJ-MSCs on lymphocyte proliferation. The results demonstrated a favorable ability of P1, P3, and P5 WJ-MSCs to inhibit lymphocyte proliferation, with percentages of 66.78 ± 6.79%, 72.72 ± 5.95%, and 70.29 ± 5.57%, respectively. Notably, no significant differences were observed between different passages, indicating that there was no decrease observed with an increase in the number of passages (*p* < 0.05) (Fig. 6C-D).

### Stability of MCB, WCB, and DP

**Fig. 7:**
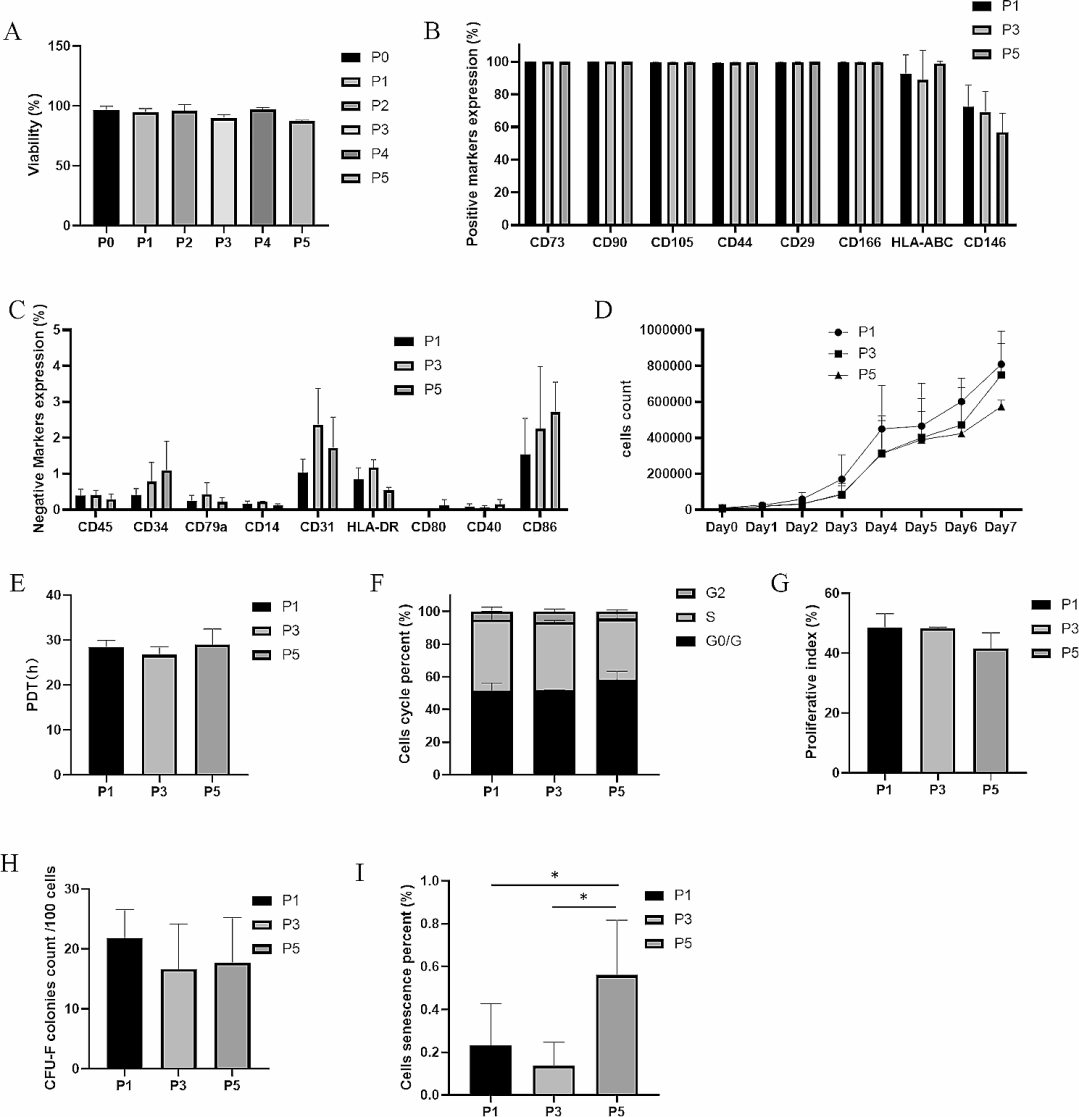
Stability study of Master Cell Bank (MCB), Working Cell Bank (WCB), and Drug Product (DP). (**A**) Cell viability changes after 1, 2, and 3 freeze-thaw cycles. (**B**) Viable cell concentration changes after 1, 2, and 3 freeze-thaw cycles. (**C**) Cell viability changes after storage at temperatures of 2–8℃ and 20–27℃ for 0, 2, 4, and 8 h. (**D**) Viable cell concentration changes after storage at temperatures of 2–8℃ and 20–27℃ for 0, 2, 4, and 8 h. ^*^p < 0.05

MCB, WCB, and DP were found to exhibit stability during storage at temperatures below − 150℃, for 6 months (Additional file (1) Table S1). DP demonstrated compliance with the specified standards for up to 2 freeze-thaw cycles. However, after undergoing 3 freeze-thaw cycles, DP failed to meet the acceptance criteria for cell viability and viable cell concentration (Additional file (2) Table S2). Notably, a significant disparity was observed between 1 and 3 freeze-thaw cycles (*p* < 0.05) (Fig. 7A-B). Following the thawing process, DP was diluted using 50mL of 0.9% sodium chloride injection and 10% HSA, and stored at different temperatures. The cell viability and viable cell concentration decreased over time, with stability observed for up to 8 h at 2–8℃, while storage at 20–27℃ did not meet the acceptance criteria after 2 h (Additional file (3) Table S3-S4 and Fig. 7C-D).

## Discussion

Currently, WJ-MSCs have been extensively utilized in clinical trials [11, 12, 39], and to meet the standards for their clinical use, a GMP-compliant manufacturing process has been documented. Studies have outlined bioprocesses for WJ-MSC production, including the establishment of MCB, WCB, and DP [27], and comparisons with BM-MSCs for identity, safety, and function [28]. A microcarrier-based bioreactor system for large-scale WJ-MSC production has also been reported [29]. In this study, we meticulously examined the development of the WJ-MSCs manufacturing process, from parameter optimization to GMP compliance. Initially, all materials selected in the production were either GMP or pharmaceutical grade. Subsequently, critical process parameters were optimized using flasks, followed by scale-up to transition to the cell factory system. Concurrently, comprehensive quality control and stability studies were conducted to ensure the generation of high-quality WJ-MSCs. Finally, we developed a streamlined and innovative approach for isolating and culturing WJ-MSCs. This method prioritizes comprehension of the process, adheres to GMP standards, and aims to enhance cell yield while maintaining the cell’s viability, growth potential, purity, and functionality throughout the manufacturing process. Ultimately, these efforts ensure the production of safe and effective WJ-MSCs.

The explant method and enzymatic digestion method are the two main approaches for isolating WJ-MSCs [20]. In this work, we conducted a parallel comparison using the same umbilical cord and the same weight of Wharton’s jelly. Our findings revealed that the enzymatic digestion method demonstrated a faster start-up, a shorter culture time, and uniform cell growth during the initial passage (P0), yet after subsequent passages, there were no significant differences between the two methods in terms of cell proliferation, cell viability, and immunophenotype. This suggests that damage caused by enzymatic digestion can be recovered during the culture process. Furthermore, the enzymatic digestion method can be easily translated into a closed and automated platform, making it more suitable for GMP-compliant processes in a standardized manufacturing setting [40, 41]. In contrast, the explant method faces difficulties in cell factory operation and may not be compatible with closed systems.

When it comes to enzymatic digestion, several factors should be considered, including the selection of enzymes, enzyme concentrations, digestion times, and seeding densities. We selected Collagenase NB6 GMP as the exclusive enzyme for WJ-MSCs isolation and digestion, thereby effectively minimizing the introduction of heterogeneous exogenous impurities and ensuring compliance with GMP regulations. In our study, we further determined the optimal enzyme concentration and digestion time for umbilical cord tissue digestion. It is worth noting that Collagenase AF-1 GMP from the same manufacturer has been launched as an animal-free alternative, which is more suitable for GMP-compliant applications.

To investigate the impact of seeding density on cell expansion, we simultaneously explored the effects of initial and passage seeding density. For the initial cell seeding density, we employed tissue fragments of different weights, which resulted in varied quantities of primary cells after digestion. These primary cells, cultured further, formed P0 cells. Our findings demonstrated a positive correlation between the weight of umbilical cord tissue and the primary seeding cell number as well as the number of P0 cells. However, no correlation was observed between the primary seeding cell number and the number of P0 cells. This discrepancy could be attributed to individual variations and the intricate composition of umbilical cord cells. In the initial stages, the presence of different cell types in the umbilical cord, such as MSCs, endothelial cells, epithelial cells, fibroblasts, and cord blood cells [42, 43], posed a challenge as only a limited number of primary cells were able to adhere and form colony-forming units (CFUs). Previous research studies have reported similar findings. One study indicated that it was 1 CFU for every 333 cells of primary human umbilical cord perivascular (HUCPV) cells [44], while another study reported a CFU-F frequency of 1:1609 in nucleated cells from the umbilical cord [45]. Consequently, we consider the weight of umbilical cord tissue as a superior parameter compared to the number of primary cells for determining the initial seeding density in the enzymatic digestion method based on our findings, which is more convenient in GMP-compliant manufacturing.

Regarding the passage seeding density, we analyzed the cell expansion at different densities. The results showed no significant differences in PDT among different densities (*p* > 0.05). However, significant differences were observed in terms of culture period, cumulative doubling times, glucose, and lactate metabolism (*p* < 0.05). It is noteworthy that utilizing a lower passaging density, as opposed to a higher passaging density, can reduce the number of passages needed to attain the desired yield. However, the overall cumulative population doublings remain consistent, whether using a lower passaging density with fewer passages or a higher passaging density with more passages. Previous studies have shown that extremely low passage densities (50–100/cm^2^) can lead to higher proliferation rates and delayed cellular senescence in BM-MSCs [46–47]. Nonetheless, the advantages observed with these low densities may not directly apply to large-scale manufacturing processes due to the risks of production failure and increased contamination associated with additional media exchanges.

In addition to the aforementioned factors, the choice of culture medium is crucial in GMP-compliant production processes. Opting for a xeno-free and serum-free medium (XF/SFM) is generally considered advantageous [48–49]. Nevertheless, most XF/SFM lack adhesive proteins, which are important for the attachment and spread of MSCs. Therefore, the use of a suitable coating solution may be necessary in such cases. However, when considering large-scale manufacturing, employing a coating solution is time-consuming and can be inconvenient [50–51]. In such scenarios, a favorable alternative to enhance MSC attachment and expansion is the addition of human platelet lysate (hPL) [19], which has been proven to be a desirable supplement for generating GMP-compliant cell products [52–55]. hPL contains a high concentration of growth factors, adhesion molecules, and chemokines, which promote MSC attachment and expansion. Compared to FBS, hPL offers several significant advantages, including enhanced proliferation behavior, reduced population doubling time, preservation of clonogenicity, increased CFU-F size, maintenance of characteristic immunophenotype, preserved in vitro trilineage differentiation capacity, maintained in vitro T-cell immunosuppression, and absence of in vivo tumorigenicity. More importantly, the use of hPL eliminates the risks associated with the transmission of animal-derived viruses [56].

In our study comparing the effects of different concentrations (2%, 5%, and 10%) of hPL on primary cell expansion, interesting outcomes were observed. The results demonstrated that both 2% and 5% concentrations showed similar levels of cell expansion. Nevertheless, using a 10% concentration resulted in decreased cell expansion. This finding is consistent with previous studies. According to Shansky et al., a 5% concentration of hPL was found to be more effective than both 1% and 10% concentrations in supporting AT-MSC growth. Similarly, Azouna et al. reported that the PDT of 5% hPL was not significantly lower than that of 10% HPL or a combination of 10% FBS and 5% hPL [57, 58]. A meta-analysis has shown that 5% hPL is superior to 10% FBS [59], and Kirsch et al. found that even a lower concentration of 2.5% hPL exhibited a higher proliferation and differentiation rate compared to 10% human serum (HS) or 10% fetal calf serum (FCS) in AT-MSCs [60]. Notably, the discussed above studies conducted their experiments by supplementing basal media, such as α-MEM or DMEM, with additional components. Moreover, it was discovered that when using defined serum-free mesenchymal stem cell media that have been optimized for growth factors, the concentration of hPL can be further reduced to 1% or even as low as 0.5% [61–62]. This observation may explain the findings of our study, suggesting that the efficacy of hPL in supporting MSC attachment and growth can be maximized even at lower concentrations when used in conjunction with serum-free media formulations.

According to a study by Ikebe et al., the use of BM-MSCs in clinical trials from 2007 to 2013 showed that 23% of trials used cells from passage 1 or less, 71% used cells from passages 1–5, and only 6% used cells from passages over 5 [63]. Sareen et al. demonstrated that an increase in passage number (from P3 to P7) in cell culture did not have a significant effect on the immune privilege of BM-MSCs [64]. However, another study found that BM-MSCs gradually lost their typical fibroblast-like spindle shape from P3 to P8, resulting in elevated morphological abnormalities and inhomogeneity. The cell population doubling rate also decreased [65]. Zhao et al. found that UC-MSCs at P3, P6, and P15 showed similar morphology, biomarker expression, and cytokine secretion. Nevertheless, the therapeutic effect on aGVHD in vivo declined at P15 [66]. Yu et al. observed that BM-MSCs grew well for 20 population doublings but experienced cellular senescence at approximately 40 PD [67]. Based on our stability study, it was found that passages 2 to 5, with a PD of less than approximately 20, were the better passages in terms of high viability and proliferation ability, particularly passages 2 or 3. For allogeneic therapy requiring an abundant number of cells, passages 4 or 5 may be the most suitable.

Noteworthy, when transitioning the culture of MSCs from flasks to cell factories, the proliferation rate tends to decrease in our study. This can be attributed to the heterogeneity of the physical and chemical environment, as well as the emergence of concentration gradients in cell factories, mainly due to gas exchange occurring at the medium/headspace gas interface [68].

However, despite the decline in the cell proliferation rate, successful scale-up of MSC manufacturing has been achieved, resulting in high-quality drug products. These included high cell viability, preservation of a consistent immunophenotype, low cellular senescence percentage, stable karyotype, and maintenance of multilineage differentiation potential. Furthermore, MSCs showed potent inhibition of lymphocyte proliferation and were free from microbial contamination. The theoretical total mean quantity of DP reached 10^13^ from one UC.

Cell surface markers are one of the key indicators used to identify MSCs. In addition to testing the marker expression defined by the International Society for Cellular Therapy (ISCT) [4], we also detected the expression of other markers, such as adhesion molecules CD44, CD29, and CD166, functional marker CD146, immunogenic markers HLA-ABC, and costimulatory molecules CD40, CD80, and CD86 in pilot-scale manufacturing. Our results revealed that CD73, CD90, CD105, CD44, CD29, and CD166 were expressed at levels higher than 95%, which are currently used to define MSCs [69]. On the other hand, CD45, CD34, CD79a, CD14, and CD31 were expressed at levels lower than 2%, implying a high MSC purity. Moreover, we found that HLA-DR and costimulatory molecules CD40, CD80, and CD86 were expressed at low levels, while HLA-ABC was expressed positively. HLA-ABC plays a role in protecting MSCs from destruction by natural killer cells, while MHC-II helps in evading immune recognition by T cells. The costimulatory molecules CD40, CD80, and CD86 are part of the second signaling system for T lymphocyte activation. These findings indicate that WJ-MSCs manufactured by our method are unlikely to trigger an immune response and can evade host immune attack in vivo [70, 71]. CD44, a receptor for hyaluronan, plays a critical role in facilitating cell migration and recruiting MSCs to wound sites for tissue regeneration [72]. CD29, also known as integrin beta-1, together with CD44, has been implicated in the processes of MSC adhesion, migration, and engraftment [73]. CD166, also known as ALCAM (Activated Leukocyte Cell Adhesion Molecule), is a cell adhesion molecule that mediates both heterophilic (ALCAM-CD6) and homophilic (ALCAM-ALCAM) cell-cell interactions. It also plays a crucial role in the migration and adhesion of MSCs [74–75]. Furthermore, CD166 serves as an important identification marker for MSCs and can effectively differentiate them from fibroblasts [76]. CD44, CD29, and CD166 were found to exhibit positive expressions in adipose as well as other tissue sources of MSCs [77], which is consistent with our findings.

Recent research has shown that CD146 is a potency marker. The CD146^+^ subpopulation has enhanced immunosuppressive capacity, resulting in improved therapeutic outcomes [78, 79]. We found that the expression of CD146 gradually decreased with increasing passages, indicating that higher passages should be unsuitable for clinical practices. Interestingly, the expression of all the mentioned markers aligns with the results of Mebarki et al. [12].

Stability study is crucial in the validation process of GMP-compliant methods, as they help identify suitable storage conditions and durations [80]. For MCB, WCB, and DP, we have observed that long-term stability during storage at temperatures below − 150℃ can be achieved for at least 6 months, and further research is continuing to determine longer durations. In clinical scenarios where DP cells are thawed but the infusion is unexpectedly canceled due to emergencies, it becomes necessary to re-cryopreserve the cells. This study demonstrates that even after undergoing 2 freeze-thaw cycles, MSCs still meet the required quality requirements, providing support for such emergencies. The choice of cell storage medium is necessary for maintaining a stable environment that significantly affects the cell viability and potency of MSCs [81]. After thawing and diluting DP cells, storing them in a 10% HSA solution at 2–8℃ can meet the criterion for up to 8 h, despite the decrease in cell viability and viable cell concentration, providing sufficient time before clinical administration.

It Is worth noting that although our study established a GMP-compliant manufacturing method, manual operations involved in extracting Wharton’s jelly and mincing the umbilical cord increase the risk of contamination. Some successful research studies have utilized the entire umbilical cord for digestion without the need to open and remove the vessels to obtain WJ-MSCs [41, 82]. However, it is important to mention that these studies were conducted using umbilical cords of approximately 1 cm in length, and the results for longer lengths are still unknown. Therefore, further optimization of the isolation method is still required to automate the process and improve consistency. Additionally, our study highlights certain limitations associated with the use of cell factories, suggesting a shift toward the use of bioreactors. Bioreactors allow for precise monitoring and tight regulation of essential culture parameters, including pH value, temperature, dissolved O_2_, and CO_2_ levels [68, 83]. This trend of utilizing bioreactors offers promising advantages in enhanced control and scalability for MSC production.

## Conclusion

In summary, our study successfully established a GMP-compliant isolation and culture method for manufacturing Wharton’s jelly-derived MSCs. We have extensively optimized the parameters and conducted scaled-up manufacturing. The resulting cell product underwent thorough evaluations for identity, purity, viability, potency, proliferative capacity, genomic stability, and microbiological safety. The establishment of this method represents a significant advancement in the field and holds great promise for clinical translation.

### Electronic supplementary material

Below is the link to the electronic supplementary material.

Supplementary Material 1

## Data Availability

All data generated during this research are included in this published article.

## Abbreviations

WJ-MSCs: Umbilical cord Wharton’s jelly derived mesenchymal stem cells
GMP: Good manufacturing practice
hPL: human platelet lysate
ISCT: International Society of Cellular Therapy
BM-MSCs: Bone marrow mesenchymal stem cells
AT-MSCs: Adipose tissue mesenchymal stem cells
SOPs: Standard operating procedures
MCB: Master cell bank
WCB: Working cell bank
PDT: Population doubling time
PDL: Population doubling level
PI: Proliferative Index
CFU-Fs: Colony-forming units-fibroblasts
PBMCs: Peripheral blood mononuclear cells
CFSE: Carboxyfluorescein diacetate succinimidyl ester
CPDs: Cumulative population doublings
PDs: Population doublings
MLR: Mixed lymphocyte reaction
FBS: Fetal bovine serum
HS: Human serum
FCS: Fetal calf serum
