# Supplementary material for: A GMP-compliant manufacturing method for Wharton’s jelly-derived mesenchymal stromal cells
Source: Stem Cell Res Ther. 2024 May 3;15:131. doi: 10.1186/s13287-024-03725-0 (PMC11069138; doi:10.1186/s13287-024-03725-0)
Supplement: Supplementary file 1 — Supplementary Material 1 [file 13287_2024_3725_MOESM1_ESM.pdf]

Additional File 1. Table S1 Long-term stability of MCB, WCB, and DP

Assay items		MCB			WCB			DP		
		0 month	3 months	6 months	0 month	3 months	6 months	0 month	3 months	6 months
Viability (%)		94.60 ± 3.23	92.62± 1.41	93.31 ± 0.77	89.70 ± 2.95	91.15 ± 1.53	89.48 ± 1.36	87.30 ± 0.81	86.39 ± 0.44	88.20 ± 0.83
Cell concentration (×10^6 mL)		5.01 ± 0.23	4.97 ± 0.12	5.05 ± 0.19	9.95 ± 0.67	9.86 ± 0.54	9.91 ± 0.39	9.79 ± 0.32	9.90 ± 0.74	9.87 ± 0.40
Surface marker expression (%)	CD90	99.83 ± 0.11	99.96 ± 0.06	99.99 ± 0.01	99.86 ± 0.06	99.83 ± 0.26	99.96 ± 0.06	99.82 ± 0.12	100.00 ± 0.00	99.99 ± 0.01
	CD105	99.68 ± 0.21	99.81 ± 0.18	99.90 ± 0.14	99.71 ± 0.19	99.45 ± 0.90	99.92 ± 0.08	99.59 ± 0.28	99.90 ± 0.14	99.84 ± 0.16
	CD73	99.75 ± 0.15	99.88 ± 0.18	99.86 ± 0.20	99.83 ± 0.07	99.88 ± 0.14	99.88 ± 0.06	99.80 ± 0.10	99.97 ± 0.05	99.82 ± 0.28
	CD166	99.72 ± 0.09	99.00 ± 1.67	99.95 ± 0.05	99.66 ± 0.23	99.66 ± 0.40	99.91 ± 0.07	99.72 ± 0.12	99.88 ± 0.21	99.97 ± 0.03
	CD45	0.40 ± 0.17	0.50 ± 0.42	0.38 ± 0.35	0.40 ± 0.13	0.48 ± 0.31	0.94 ± 0.21	0.28 ± 0.15	0.49 ± 0.27	0.16 ± 0.09
	CD34	0.41 ± 0.17	0.69 ± 0.14	0.71 ± 0.13	0.78 ± 0.53	0.93 ± 0.03	0.96 ± 0.15	1.08 ± 0.82	0.76 ± 0.05	0.83 ± 0.19
	HLA-DR	0.84 ± 0.33	0.99 ± 0.17	0.73 ± 0.10	1.17 ± 0.22	0.82 ± 0.12	0.83 ± 0.07	0.54 ± 0.08	0.93 ± 0.14	0.75 ± 0.22

CD14	0.17 ± 0.07	0.50 ± 0.39	0.32 ± 0.38	0.22 ± 0.02	0.53 ± 0.40	0.84 ± 0.28	0.12 ± 0.05	0.35 ± 0.26	0.10 ± 0.07
CD79a	0.24 ± 0.16	0.72 ± 0.21	0.58 ± 0.17	0.43 ± 0.31	0.86 ± 0.11	0.73 ± 0.12	0.21 ± 0.13	0.80 ± 0.16	0.54 ± 0.26
Sterility	Negative	Negative	Negative	Negative	Negative	Negative	Negative	Negative	Negative
Mycoplasma	Negative	Negative	Negative	Negative	Negative	Negative	Negative	Negative	Negative
Endotoxin	<0.5EU/mL	<0.5EU/mL	<0.5EU/mL	<0.5EU/mL	<0.5EU/mL	<0.5EU/mL	<0.5EU/mL	<0.5EU/mL	<0.5EU/mL
Lymphocyte proliferation inhibition (%)	66.78 ± 6.79	53.54 ± 10.25	60.37 ± 26.63	72.72 ± 5.95	53.61 ± 10.14	58.47 ± 13.17	70.29 ± 5.57	71.45 ± 16.86	76.73 ± 4.57
Multilineage differentiation potential	Positive	Positive	Positive	Positive	Positive	Positive	Positive	Positive	Positive

Additional File 2. Table S2 Multiple freeze-thaw stability of DP

Assay items		Acceptance criteria	Batch 1			Batch 2			Batch 3		
			1 cycle	2 cycles	3 cycles	1 cycle	2 cycles	3 cycles	1 cycle	2 cycles	3 cycles
Viability (%)		≥80	84.8	89.4	75.5	94.5	88.6	89.9	91.0	82.9	83.9
Viable cell concentration ($\times 10^6$ /mL)		≥0.93	1.01	1.07	0.81	1.01	0.94	0.88	1.05	0.88	0.87
Surface marker expression (%)	CD90	≥95	100.00	99.95	99.96	100.00	100.00	99.97	100.00	100.00	99.98
	CD105	≥95	99.98	99.91	99.43	99.93	99.64	99.90	99.97	99.59	99.96
	CD73	≥95	100.00	99.63	99.91	99.98	99.83	99.92	99.95	99.94	99.93
	CD166	≥95	99.98	99.89	99.87	99.99	99.85	99.97	100.00	99.92	99.98
Sterility		Negative	Negative	Negative	Negative	Negative	Negative	Negative	Negative	Negative	Negative

Additional File 3. Table S3 In-use stability of DP at 2-8 °C

Assay items		Acceptance criteria	Batch 1				Batch 2				Batch 3			
			0 h	2 h	4 h	8 h	0h	2 h	4 h	8 h	0h	2 h	4 h	8 h
Viability (%)		≥80	93.0	92.4	89.8	87.9	93.1	91.8	91.5	85.1	92.0	89.0	89.1	86.4
Viable cell concentration ($\times10^6$ /mL)		≥0.66*	0.82	0.79	0.72	0.68	0.82	0.85	0.87	0.73	0.86	0.79	0.84	0.72
Surface marker expression (%)	CD90	≥95	99.98	99.90	99.95	99.76	100.00	100.00	100.00	99.99	100.00	99.97	99.95	99.96
	CD105	≥95	99.91	99.90	99.93	99.86	99.95	99.28	99.83	96.81	99.99	99.96	99.91	99.88
	CD73	≥95	99.98	99.80	99.94	99.85	99.95	99.99	99.98	99.98	99.96	100.00	99.88	99.95
	CD166	≥95	99.98	99.91	99.93	99.86	99.82	99.93	99.31	99.92	99.99	99.97	99.97	99.90
Sterility		Negative	Negative	Negative	Negative	Negative	Negative	Negative	Negative	Negative	Negative	Negative	Negative	Negative

*80% of the theoretical concentration after dilution with 50mL of 0.9% sodium chloride injection and 10% human serum albumin.

Additional File 3. Table S4 In-use stability of DP at 20-27 °C

Assay items		Acceptance criteria	Batch 1				Batch 2				Batch 3			
			0 h	2 h	4 h	8 h	0h	2 h	4 h	8 h	0h	2 h	4 h	8 h
Viability (%)		≥80	91.8	87.8	83.9	70.0	92.5	92.1	88.4	84.1	88.7	84.3	80.9	65.9
Viable cell concentration ($\times 10^6$ /mL)		≥0.66*	0.84	0.72	0.43	0.51	0.85	0.86	0.80	0.76	0.90	0.80	0.74	0.57
Surface marker expression (%)	CD90	≥95	99.98	99.97	99.93	99.85	100.00	100.00	100.00	100.00	100.00	99.94	99.98	99.85
	CD105	≥95	99.91	99.92	99.90	95.98	99.95	99.77	99.94	99.90	99.99	99.87	99.92	99.65
	CD73	≥95	99.98	99.89	99.84	97.37	99.99	99.96	99.96	99.98	99.90	99.91	99.83	99.98
	CD166	≥95	99.98	99.94	99.88	95.34	100.00	99.97	99.98	99.95	99.90	99.87	99.83	100.00
Sterility		Negative	Negative	Negative	Negative	Negative	Negative	Negative	Negative	Negative	Negative	Negative	Negative	Negative

*80% of the theoretical concentration after dilution with 50mL of 0.9% sodium chloride injection and 10% human serum albumin.
